# Enhanced Antitumor Effects of Adenoviral-Mediated siRNA against GRP78 Gene on Adenosine-Induced Apoptosis in Human Hepatoma HepG2 Cells

**DOI:** 10.3390/ijms15010525

**Published:** 2014-01-03

**Authors:** Ling-Fei Wu, Yi-Tian Guo, Qing-Hua Zhang, Meng-Qi Xiang, Wei Deng, Yan-Qing Ye, Ze-Jin Pu, Jia-Lin Feng, Guan-You Huang

**Affiliations:** 1 Department of Gastroenterology and Information, Second Affiliated Hospital, Shantou University Medical College, Shantou 515041, China; E-Mails: yitian7000@163.com (Y.-T.G.); zhangqinghua1210@163.com (Q.-H.Z.); xiangmengqi2362@126.com (M.-Q.X.); 07wdeng1@stu.edu.cn (W.D.); gnmu2002@126.com (Y.-Q.Y.); puzejin@sina.com (Z.-J.P.); jlfeng6@tom.com (J.-L.F.); 2 Reproductive Medicine Center, Affiliated Hospital of Guiyang Medical College, Guiyang 550004, China; E-Mail: guanyouhuang@hotmail.com

**Keywords:** ER stress, glucose-regulated protein 78 (GRP78), adenosine, RNA interference, caspase-4

## Abstract

Our previous studies show that adenosine-induced apoptosis is involved in endoplasmic reticulum stress in HepG2 cells. In this study, we have investigated whether knockdown of GRP78 by short hairpin RNA (shRNA) increases the cytotoxic effects of adenosine in HepG2 cells. The adenovirus vector-delivered shRNA targeting GRP78 (Ad-shGRP78) was constructed and transfected into HepG2 cells. RT-PCR assay was used to determine RNA interference efficiency. Effects of knockdown of GRP78 on adenosine-induced cell viabilities, cell-cycle distribution and apoptosis, as well as relative protein expressions were determined by flow cytometry and/or Western blot analysis. The intracellular Ca^2+^ concentration was detected by laser scanning confocal microscope. Mitochondrial membrane potential (ΔΨm) was measured by a fluorospectrophotometer. The results revealed that GRP78 mRNA was significantly downregulated by Ad-shGRP78 transfection. Knockdown of GRP78 enhanced HepG2 cell sensitivity to adenosine by modulating G0/G1 arrest and stimulating Bax, Bak, m-calpain, caspase-4 and CHOP protein levels. Knockdown of GRP78 worsened cytosolic Ca^2+^ overload and ΔΨm loss. Knockdown of caspase-4 by shRNA decreased caspase-3 mRNA expression and cell apoptosis. These findings indicate that GRP 78 plays a protective role in ER stress-induced apoptosis and show that the combination of chemotherapy drug and RNA interference adenoviruses provides a new treatment strategy against malignant tumors.

## 1. Introduction

Hepatocellular carcinoma (HCC) is the fifth most common cancer and the third leading cause of cancer-related deaths worldwide [1]. Treatment options include surgery, chemotherapy, and interventional radiation therapy alone and in combination. HCC is still associated with poor prognosis, with only approximately 30% of diagnosed patients being considered eligible for potential curative treatment. Chemotherapy and interventional therapy are mainly used for advanced tumors. The major clinical problems include resistance to current therapy, elevated toxicity of standard drugs, and the repopulation of cells that escape chemotherapy [2]. Therefore, the development of novel strategies aimed at improving the chemotherapeutic effects of hepatocellular carcinoma is of significant importance.

Apoptosis-based anticancer therapies require an in-depth understanding of the apoptotic components of malignant cells, including apoptotic regulators and apoptotic executioners, because their expression levels and functional integrity determine the efficacy of therapy [3]. Apoptosis is initiated in a caspase-dependent and -independent manner. Death receptors, mitochondria and endoplasmic reticulum (ER) engage either extrinsic or intrinsic caspase activation. The intrinsic mitochondrial pathway relies on the caspase-9 as initiator caspase and is controlled by Bcl-2 family members. The extrinsic pathway relies on caspase-8 and −10 as initiator caspases, is triggered by direct engagement of cell-surface death receptors, and can either converge on mitochondria or circumvent mitochondria to directly activate effector caspases [4]. Adenosine, a ubiquitous metabolite from ATP hydrolysis, induces apoptosis in a variety of cancer cells and is a chemotherapy drug with wide prospect of clinical application [5–8]. In our previous studies, adenosine induces apoptosis in EC 109 and HepG2 cells by the ER stress pathway [7,9]. However, the potential roles of ER stress-related genes in the cytotoxic effects of adenosine are unclear.

The endoplasmic reticulum (ER) is the primary subcellular organelle where proteins are synthesized and folded. Only properly folded (mature) proteins can be transported to the Golgi apparatus for further processing. The ER also serves as a dynamic pool of Ca^2+^, maintaining intracellular Ca^2+^ homeostasis [10]. When the homeostasis of the ER is disturbed, unfolded or misfolded proteins accumulate in the ER lumen, resulting in ER stress. In response to ER stress, cells activate a cascade of recovery actions, collectively named the unfolded protein response (UPR). The UPR is a series of complementary adaptive mechanisms to cope with protein-folding alterations [11]. The UPR reestablishes homeostasis and normal ER function in the cell by blocking protein translation and activating the signaling pathways that lead to increasing production of molecular chaperones involved in protein folding. However, when adaptive mechanisms fail to restore normal ER function due to protracted or excessive stress stimuli, the UPR pathways can initiate apoptotic pathways to remove the stressed cells [12]. There are three branches of the UPR that are initiated by distinct ER stress transducers located on the ER membrane: PERK, IRE-1a and ATF6 [13]. In unstressed cells, all three ER stress transducers are kept in an inactive state through binding to the ER chaperone glucose-regulated protein 78 (GRP78), which is also known as immunoglobulin binding protein (Bip). Upon ER stress, excessive unfolded proteins accumulate in the ER lumen, resulting in the dissociation of GRP78 from the ER stress transducers, which triggers activation of the UPR branches. The duration of UPR activation determines the effects on cell fate: in the early stages it induces cell survival and increases refolding activity within the ER by activating the ATF6 and IRE-1a branches, whereas prolonged activation results in apoptosis [14]. Several mechanisms link ER stress to apoptosis, including C/EBP homologous protein (CHOP), c-JUN NH2-terminal kinase (JNK) and caspase-4 activation, the latter which can initiate a specific mitochondria-independent caspase (caspase-8, −9) cascade [3,4,15]. Cross-talk with mitochondria is mediated by various factors, including Ca^2+^, Bcl-2 family proteins, and stress-associated kinases [16]. GRP78 is a chief regulator of ER function and is overexpressed in several clinically refractory tumors, such as glioma [17], leukemia [18], and breast cancer [19]. Increased GRP78 levels are also associated with poor outcome and early recurrence in prostate cancer [20]. Conversely, GRP78 insufficiency in mouse cancer models slows the progression of tumors [21]. GRP78 up-regulation in various tumor types and its induction after drug treatment, have been shown to be major contributors to tumorigenesis and therapeutic resistance [14,22]. Thus, therapies aimed at decreasing GRP78 levels, which result in the inhibition of tumor cell proliferation and resensitization of tumor cells to chemotherapeutic drugs, may hold promise for cancer treatment.

Small interfere RNAs, such as micro inferring (miRNA) or short hairpin RNAs (shRNAs), endogenously suppress gene expression and hundreds of miRNAs have been described [23]. RNAi can act as oncogenes or as tumor suppressors by targeting molecules critically involved in carcinogenesis. However, the specific short hairpin RNA of GRP78 has yet to be characterized. Based on these considerations, we construct a recombinant adenovirus containing short hairpin RNA targeting GRP78 and determine whether GRP78 plays a role in the chemoresistance of hepatocellular cancers and if so, the possible ER stress-relative molecular mechanisms.

## 2. Results and Discussion

### 2.1. Results

#### 2.1.1. Verification of GRP78 Gene Knockdown and Evaluation of the Effects on Cell Growth

**Figure 1.**
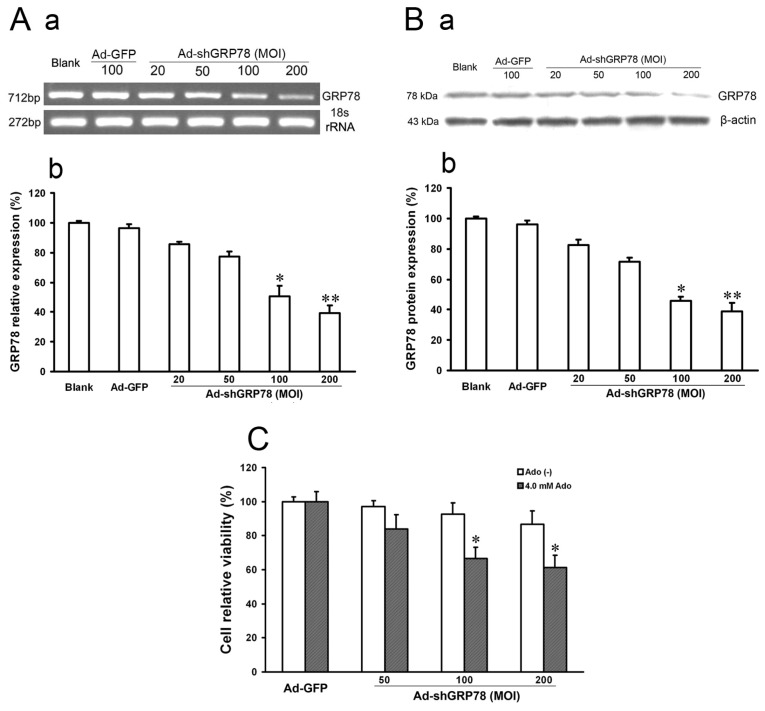
The effects of recombinant adenovirus vector (Ad-shGRP78) on the mRNA and protein expressions of GRP78 and cell growth. Adenoviral vector Ad-shGRP78 was successfully constructed according to the instructions mentioned before. HepG2 cells were infected with different MOIs (20, 50, 100, 200). GRP78 mRNA expression was analyzed by semi-quantitive real-time reverse transcriptional PCR (RT-PCR, **A-a**); Band intensity analysis was performed with the Quantity One software (**A–b**); Similarly, GRP78 protein expression was analyzed by western blot (**B-a**); Band intensity analysis was also performed with the Quantity One software (**B-b**); HepG2 cells were infected with adenoviral vectors and incubated with or without 4.0 mmol/L adenosine for 24 h, and cell viability was determined by the CCK-8 assay (**C**). Each value represents the mean ± SD of three independent experiments. * *p* < 0.05, ** *p* < 0.01, *vs.* untreated control or negative control group.

In the present study, we described the generation of a recombinant adenovirus containing short hairpin RNA targeting GRP78 (Ad-shGRP78). The pcDNA6.2-GW/EmGFP-GRP78-miR plasmid vectors and the recombinant adenovirus vectors (Ad-shGRP78) were identified using sequencing. The particular sequence of RNAi insertion into vectors was the same as designed. While Ad-GFP or Ad-shGRP78 was transfected into 293A cells on the 13th day, nearly 100% GFP were expressed in fluorescence states, suggesting that the recombinant adenovirus vectors were successfully constructed and effectively transfected into 293A cells. Immune assay was utilized to titre the virus. The RNAi adenovirus vector (Ad-shGRP78) was calculated as 1.1 × 10^9^ pfu/mL and the empty adenovirus vector (Ad-GFP) was 1.4 × 10^9^ pfu/mL. The inhibition effects of Ad-shGRP78 on GRP78 mRNA and protein were observed by RT-PCR and western blot assay, respectively. As shown in Figure 1A, compared with the blank group or negative control group (Ad-GFP group), the mRNA expression of GRP78 was significantly decreased to 24.5% and 19.3% after Ad-shGRP78 transfection at 100 and 200 MOI (*p* < 0.01), showing a marked dose-dependent inhibition of GRP78 expression. Western blot assay showed similar results (Figure 1B), which suggests effective GRP78 inhibition by Ad-shGRP78 in HepG2 cells. As a direct test of whether GRP78 protects HepG2 cells against adenosine-induced cell death, CCK-8 was used to detect cell viability. Adenovirus infection at MOIs of 50, 100 and 200, compared with the control group, did not alter cell viability (*p* > 0.05), suggesting that knockdown GRP78 does not affect HepG2 cell proliferation under a no adenosine treatment condition (Figure 1C). However, knockdown of GRP78 significantly enhanced adenosine-induced growth inhibition in a dose-dependent manner at 100 and 200 MOI (66.65% ± 8.58% and 61.36% ± 7.13% *vs.* 100% ± 8.89% compared with the positive control, Figure 1C, *p* < 0.05), indicating that knockdown GRP78 can not affect HepG2 cell growth under normal physiological conditions, but enhances the cytotoxic effects of adenosine. In other words, GRP78 plays a protective role in ER stress.

#### 2.1.2. Effects of GRP78 Knockdown on Adenosine-Induced Alterations in Cell-Cycle Distribution and Apoptosis

**Figure 2.**
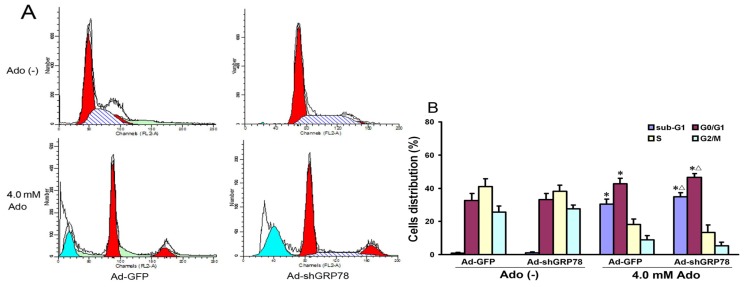
Effects of GRP78 knockdown on cell cycle distribution and apoptosis (sub-G1). HepG2 cells were transfected with either Ad-GFP (negative control) or Ad-shGRP78. After incubation for 24 h, cells were incubated with or without 4.0 mmol/L adenosine for an additional 24 h, and the cell cycle phases were determined by propidium iodide staining, followed by FACS flow cytometric analysis (**A**); The values represent the means ± SD of three independent experiments (**B**). * *p* < 0.05, *vs.* negative control group (vector only treatment); ^Δ^ *p* < 0.05, *vs.* the positive control group (adenosine treatment).

Since overexpression of GRP78 in cancer cells can inhibit apoptosis [14], we next evaluated whether GRP78 knockdown affects adenosine-induced cell cycle progression and apoptosis in HepG2 cells. Flow cytometry analysis showed that adenosine treatment increased the ratios in the sub-G1 (apoptotic peak) and G0/G1 phase and decreased those in S and G2/M phases (Figure 2A). Compared with the control group, there were significant increases in both G0/G1 and sub-G1 phases (42.61% ± 5.38% *vs.* 32.64% ± 4.21%, *p* < 0.05; 30.31% ± 3.03% *vs.* 0.92% ± 0.35%, *p* < 0.01). Knockdown of GRP78 further increased the ratios of sub-G1 and G0/G1 phase cells (*p* < 0.05; Figure 2B), showing GRP78 knockdown arrests the cell cycle in the G0/G1 phase.

**Figure 3.**
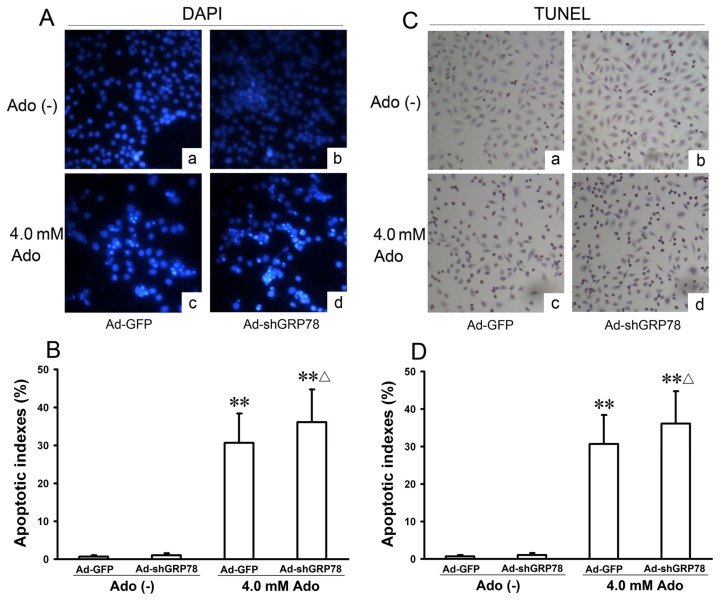
Effects of GRP78 knockdown on adenosine-induced apoptosis in HepG2 cells. HepG2 cells were transfected with either Ad-GFP (control) or Ad-shGRP78. After incubation for 24 h, cells were incubated with or without 4.0 mmol/L adenosine for a further 24 h, then cell apoptosis was examined. The morphology of apoptotic nuclei was observed by DAPI staining and fluorescence microscopy (**A**); Apoptosis was detected by TUNEL assay in HepG2 cells (**C**); The apoptotic fraction was analyzed by flow cytometry. Quantitative analysis of the total apoptotic population is presented. Each bar corresponds to the mean±SD of three independent experiments by above 2 methods (**B**,**D**). ** *p* < 0.01, *vs.* negative control group. ^Δ^ *p* < 0.05, *vs.* positive control group.

To confirm the flow cytometry analysis results, DAPI staining and TUNEL were performed. The morphologic hallmarks of apoptosis include chromatin margination, nuclear condensation and fragmentation. Normal cell nuclei were uniform and without condensation or fragmentation in the control group (Figure 3A-a). In HepG2 cells treated with adenosine or co-treated with Ad-shGRP78 and adenosine, cell nuclei became condensed and shrunken; typical apoptotic bodies appeared (Figure 3A–c and 3A–d). Both cell apoptotic ratios in adenosine alone and the combination treatment group by DAPI staining were over 40-fold higher than that in control group (30.70% ± 7.66%, 36.10% ± 8.68% *vs.* 0.74% ± 0.26%; both *p* < 0.01); and the apoptotic ratio in combination treatment group was higher than that in adenosine alone group (*p* < 0.05, Figure 3B). TUNEL assay also showed the similar results (Figure 3C,D). These data are consistent with previous studies of cell growth inhibition, indicating that adenosine-mediated growth inhibition is at least partly due to the G0/G1 phase arrest and apoptosis induction. These results further demonstrate that knockdown of GRP78 enhances the cytotoxicity of adenosine in HepG2 cells.

#### 2.1.3. Effects of Caspase-4 Knockdown on Adenosine-Induced Cell Growth Inhibition and Apoptosis

**Figure 4.**
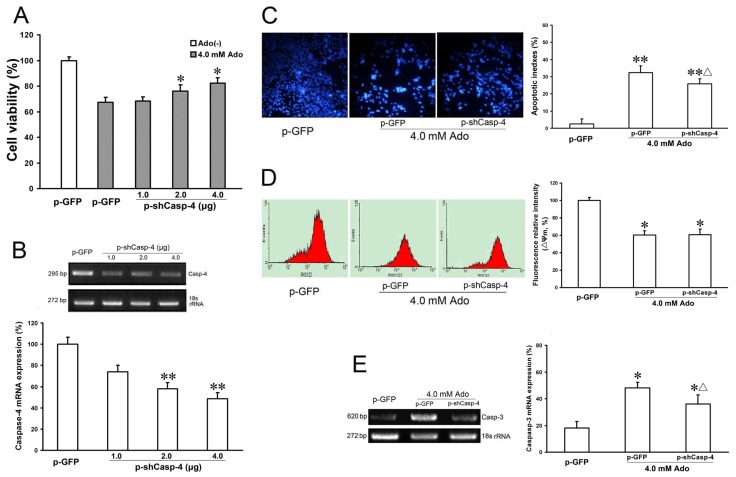
Effects of p-shCasp-4 on caspase-4 mRNA expression and ER stress-related proteins in adenosine-induced apoptosis. HepG2 cells were transfected with either p-GFP (control) or 1.0, 2.0 or 4.0 μg DNA of p-shCasp-4. After incubation for 24 h, cells were incubated with or without 4.0 mmol/L adenosine for a further 24 h, and cell viability was determined by CCK-8 assay (**A**); HepG2 cells were transfected as in panel A. GRP78 mRNA expression was analyzed by RT-PCR. p-shCasp-4 groups showed caspase-4 mRNA expression was down-regulated with increasing doses of plasmid vectors (**B**); HepG2 cells were transfected with either p-GFP (control) or p-shCasp-4. After incubation for 24 h, cells were incubated with or without 4.0 mmol/L adenosine for a further 24 h. The morphology of apoptotic nuclei was observed by DAPI staining and fluorescence microscopy. Knockdown of caspase-4 significantly reduced adenosine-induced apoptosis (**C**); HepG2 cells were transfected as in panel C. Mitochondrial membrane potential (ΔΨm) was measured by flow cytometry (**D**) and caspase-3 mRNA expression was analyzed by semi-quantitative real time PCR (**E**). The band intensity analysis was performed with Quantity One software. Data are presented as means ± SD of at least three independent experiments. * *p* < 0.05, ** *p* < 0.01, *vs.* negative control group; ^Δ^ *p* < 0.05,*vs.* positive control group.

Caspase-4 is a member of the inflammatory caspase group, and is localized to the ER. It is specifically activated by ER stress, including disruption of ER calcium homeostasis and accumulation of excess proteins in the ER. Activated caspase-4 directly cleaves procaspase-9 into active caspase-9, which further cleaves and activates caspase-3, resulting in apoptosis [15]. To identify the involvement of caspase-4 in adenosine-mediated cell death, we examined the effects of caspase-4 knockdown, by plasmid-delivered caspase-4 shRNA (p-shCasp-4), on adenosine-induced apoptosis. Real-time PCR showed that knockdown of caspase-4 with p-shCasp-4 at 2.0 and 4.0 μg DNA effectively decreased caspase-4 mRNA expression to 28.15% ± 5.65% and 23.41% ± 6.12%, respectively, compared with the control group (100% ± 6.3%, *p* < 0.05, Figure 4B). Concomitantly, knockdown of caspase-4 at 2.0 and 4.0 μg significantly increased the cell viability (78.1% ± 9.3%, 87.4% ± 7.65% *vs.* 65.3% ± 3.8% compared with the positive control, respectively, *p* < 0.05, Figure 4A). Knockdown of caspase-4 significantly decreased caspase-3 mRNA expression (*p* < 0.05, Figure 4E) and cell apoptosis (25.84% ± 4.98% *vs.* 32.40% ± 3.87%, *p* < 0.05, Figure 4C), but did not further decrease mitochondrial membrane potential (ΔΨm) (compared with the positive control, *p* > 0.05, Figure 4D) after adenosine treatment. Altogether, these results show that caspase-3 activation, but not ΔΨm is involved in caspase-4 activation, which suggests that caspase-4 plays a key role in ER stress-mediated apoptosis.

#### 2.1.4. Effects of GRP78 Knockdown on Adenosine-Induced Cytosolic [Ca^2+^]i and Mitochondrial Membrane Potential (ΔΨm)

**Figure 5.**
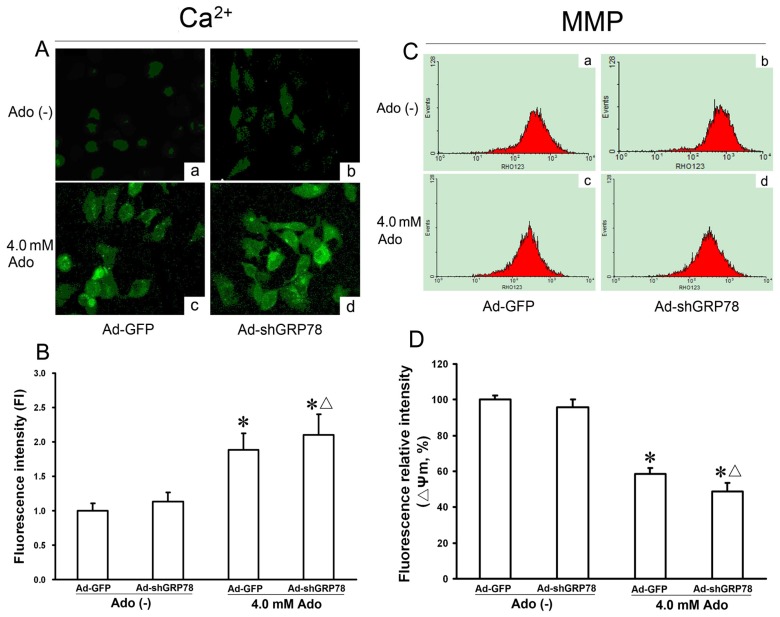
Effects of GRP78 knockdown on Ca^2+^ concentration and mitochondrial membrane potentials (ΔΨm) in adenosine-treated HepG2 cells. HepG2 cells were transfected with either Ad-GFP (control) or Ad-shGRP78. After incubation for 24 h, cells were incubated with or without 4.0 mmol/L adenosine for a further 24 h. Fluo-3/AM fluorescence and laser scanning confocal microscopy (LSCM) were utilized to measure cytosolic Ca^2+^. The relative fluorescence intensity values represent cytosolic Ca^2+^ content (**A**); ΔΨm was measured by flow cytometry (**C**); Adenosine caused a significant increase of [Ca^2+^]i and loss of ΔΨm; GRP78 knockdown further worsened the Ca^2+^ overload and loss of ΔΨm; and (**B**,**D**) Data are presented as means ± SD of at least three independents experiments. * *p* < 0.05, *vs.* the negative control group. ^Δ^ *p* < 0.05, *vs.* the positive control group.

To determine whether adenosine influences intracellular [Ca^2+^]i, the level of cytosolic Ca^2+^ was measured with Fluo-3/AM staining by LSCM. Compared with the untreated control (Ad-GFP), knockdown of GRP78 did not affect the change in fluorescence intensity (ΔF/F0) (*p* > 0.05, Figure 5A-a and 5A–b). However, treatment of HepG2 cells with adenosine alone or combination treatment with Ad-shGRP78 increased the fluorescence ratio (Δ*F*/*F*_0_) by 1.88- and 2.10-fold as compared with the control group (*p* < 0.05, Figure 5B), suggesting that adenosine but not Ad-shGRP78 increases the cytosolic [Ca^2+^]i. In other words, adenosine treatment causes ER stress and increases in cytosolic [Ca^2+^]i. Knockdown of GRP78 by RNAi reinforces the trend when HepG2 cells are treated with adenosine.

Mitochondria are an integral part of the apoptotic machinery, and the loss of ΔΨm is often caused by cytosolic Ca^2+^ overload and is a classical evidence for apoptosis. To explore the involvement of mitochondria membrane potential, ΔΨm was measured using multicolor flow cytometry. As shown in Figure 5D, compared with the control, adenosine decreased the fluorescence intensity of Rh123 from 100% ± 8.43% to 58.54% ± 8.21% (*p* < 0.05, Figure 5D). GRP78 knockdown further decreased ΔΨm to 34.78% ± 4.58%, with the difference between the adenosine group and combination treatment group being significant (*p* < 0.05, Figure 5D), indicating that GRP78 knockdown further exacerbates adenosine-induced mitochondrial member injury.

#### 2.1.5. Effects of GRP78 Knockdown on the mRNA and Protein Expressions of ER Stress Relative Genes

**Figure 6.**
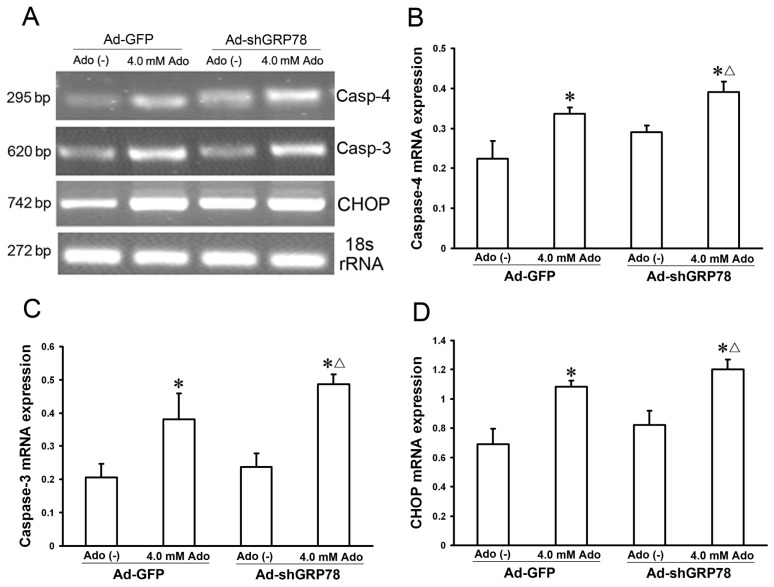
Effects of GRP78 knockdown on mRNA expressions of caspase-3, and −4, and CHOP. HepG2 cells were transfected with either Ad-GFP (control) or Ad-shGRP78. After incubation for 24 h, cells were incubated with or without 4.0 mmol/L adenosine for an additional 24 h. The cells were transfected with adenoviral vectors and without adenosine treatment as a negative control. The cells were transfected with Ad-GFP and with adenosine treatment as a positive control. Real-time PCR was used to detect mRNA expression (**A**); Adenosine caused increases in mRNA expression for all three genes. GRP78 knockdown further enhanced their mRNA expression following adenosine treatment (**B**–**D**). Data are presented as means ± SD of three independents experiments. * *p* < 0.05 *vs.* the negative control group. ^Δ^ *p* < 0.05, *vs.* the positive control group.

Caspase-4 and CHOP are two key molecules in the three branches of ER stress-associated apoptosis. GRP78 is known to interact with procaspase-4 and suppress its activation [3,15]. To investigate the effects of GRP78 on ER stress relative proteins, semi-quantitative real time PCR was used to detect their mRNA expression. The results indicated that knockdown of GRP78 results in increases in mRNA expression for caspase-4, CHOP, and caspase-3 in adenosine-treated HepG2 cells (Figure 6B–D).

**Figure 7.**
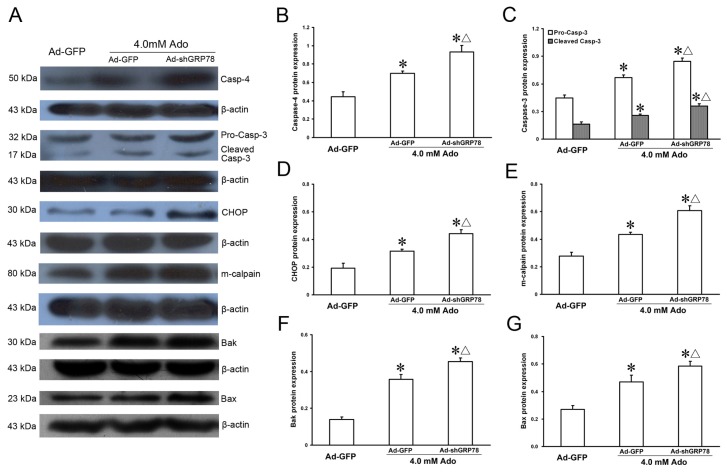
Effects of GRP78 knockdown on protein expressions of ER stress-related genes. HepG2 cells were transfected with either pAd-EGFP or Ad-shGRP78. After incubation for 24 h, cells were incubated with 4.0 mmol/L adenosine for a further 24 h. Cells transfected with Ad-GFP and without adenosine treatment served as a negative control. Protein expression of caspase-4, caspase-3, cleaved-caspase-3, CHOP, m-calpain, Bak, and Bax, were assessed by western blot (**A**); Adenosine caused protein expression increases in all ER stress-related genes examined. GRP78 knockdown further enhanced their protein expression (**B**–**G**). Data are presented as means ± SD of three independent experiments. Band intensity analysis was performed with Quantity One Software. * *p* < 0.05, *vs.* the negative control group; ^Δ^ *p* < 0.05, *vs.* the positive control group.

The protein expression levels of ER stress-related genes were also investigated by western blot and the results were similar to that of mRNA expression (Figure 7A). As shown in Figure 7B–G, knockdown GRP78 led to dramatic protein expression increases in m-calpain, Bax, Bak, caspase-4, CHOP, procaspase-3 and cleaved-caspase-3 following addition of adenosine (all *p* < 0.05, Figure 7B). These results reveal that GRP78 can suppress CHOP, caspase-4 and Bcl-2 family member induction, and that the three signaling pathways participate in ER stress-mediated cell apoptotic death.

### 2.2. Discussion

One characteristic feature of cancer cells is their ability to develop resistance to chemotherapeutic agents. GRP78 is overexpressed in some cancers refractory to therapy [17,19]. It has been shown that GRP78 induces chemoresistance in brain endothelial cells, favoring tumor vascularization and metastatic spread [24], and that GRP78 inhibition resensitizes acute lymphoblastic leukemia cells refractory to vincristine [18]. In the present study, we show a recombinant RNAi adenoviral vector (Ad-shGRP78) encoding a GFP78-targeting shRNA, decreases GFP78 mRNA levels to 24.5% and 19.3% at 100 and 200 MOI in 293A cells (Figure 1A). Our previous studies show adenosine induces HepG2 and EC109 cell apoptosis by ER stress and upregulates GRP78 expression [7,9]. Because GRP78 protects tumor cells and endows them with increased chemoresistance, downregulation of GRP78 may become an important adjunct in future anti-tumor therapy. In this study, knockdown GRP78 by RNAi not only results in adenosine-induced cell growth inhibition, but also increases adenosine-induced cell apoptosis (Figure 3). This GRP78 knockdown-mediated increase in chemosensitivity suggests inhibition of GRP78 might play a protective role in anti-tumor chemotherapy [17–19]. However, cytotoxicity following GRP78 knockdown is not evident under nonstressed conditions. These results are similar to Yung *et al.*, who show GRP78 knockdown does not affect cell proliferation under a physiological condition but sensitizes JEG-3 cells to tunicamycin [25]. We think that the modulation of GRP78 in cells is quite complex and might be dependent on cell types, stimuli and the degree of ER stress.

In our previous study, adenosine induces ER stress and NF-κB activation [7,9,26]. In this study, we show that adenosine up-regulates the expressions of Bak and Bax and causes cell accumulation in the G0/G1 phase and apoptosis (Figures 2 and 7). Knockdown of GRP78 enhances sensitivity to adenosine in HepG2 cells (Figure 3). Bcl-2 family proteins play essential roles in regulating apoptosis. Although anti-apoptotic family members (Bcl-2, Bcl-X_L_) and multidomain pro-apoptotic members (Bak, Bax) are thought to function mainly on mitochondria, recent studies suggest that they may also function on the ER where they reside as well [27]. Knockdown of GRP78 enhances cell cycle arrest in G0/G1 phase and exacerbates adenosine-induced cell apoptosis, concomitant with the increased expressions of Bak and Bax, suggesting that Bcl-2 family members may contribute to regulating ER stress-induced apoptosis [28].

Under normal ER Ca^2+^ conditions, the ER stress sensors are bound and inactivated by GRP78. Once the ER Ca^2+^ decreases, chaperone function becomes disturbed and unfolded proteins accumulate and bind GRP78. As a consequence, ER-stress sensors become unbound to GRP78 and activated, causing UPR [3,4]. Overexpression of Bcl-2 [29] or knockdown of both Bak and Bax [30] are reported to cause ER Ca^2+^ depletion, whereas Bcl-2 enhances the retention of Ca^2+^ in the ER lumen [31]. Cells deficient in Bax and Bak are resistant to ER stress-induced apoptosis [32], demonstrating the important role of Bax and Bak in ER stress. In view of the finding that adenosine decreased Bcl-2 levels in our previous study [26] and knockdown GRP78 increases Bax and Bak levels in the present study, we suggest that the reduced Bcl-2/Bax ratio causes ER Ca^2+^ depletion, leading to cytosolic Ca^2+^ overload and the loss of ΔΨm. During ER stress, activated CHOP further aggravates cell apoptotic death by reducing the Bcl-2/Bax ratio [33], and increased Bax and Bak levels in turn exacerbate the UPR by activation of the IRE-1a pathway [34].

CHOP and caspase-4 are two major pro-apoptotic genes in ER stress-induced apoptosis [15,32]. All three branches of the UPR regulate the activation of CHOP. Caspase-4, a protein predominantly localized in the ER, is specifically cleaved by ER stress but not by other non-ER stimuli, and several studies have identified caspase-4 as a key player in the ER stress-mediated pathway of apoptosis in humans or caspase-12 in rodents [13]. Caspase-4 was found localized to the ER membrane and activated specifically by ER stress-inducers. Upon activation by ER stress, the cytosolic domain of IRE1a recruits TNF receptor-associated factor 2 (TRAF2), which interacts with caspase-4 and induces the cleavage and activation of caspase-4. Activated caspase-4 cleaves procaspase-9 into active caspase-9, which further cleaves and activates caspase-3, resulting in apoptosis. In caspase-4-mediated apoptotic process, cytochrome c is not released from mitochondria, which suggest that cytochrome c is not involved in the caspase-4-dependent apoptosis [35]. Knockdown of caspase-4 gene by siRNA significantly reduces NF-κB activation and nuclear translocation [36]. Our previous studies show that adenosine induces NF-κB activation in EC109 cells [7] and HepG2 cells [26]. In the present study, we have demonstrated that knockdown of caspase-4 decreases both adenosine-induced activation of caspase-3 and apoptotic cell death, suggesting that caspase-3, the central effecter of apoptosis, acts downstream from caspase-4 in the ER stress-induced apoptotic pathway. Caspase-4-mediated caspase-3 activation may not involve the mitochondria, since ΔΨm does not change after knockdown of caspase-4. GRP78 knockdown increases sensitivity to induction of Bax, Bak and m-calpain levels by apoptotic stimuli, causing intracellular Ca^2+^ overload, ΔΨm loss and cell cycle arrest, as well as activating the caspase-4 and CHOP signal pathways and their downstream substrates. Knockdown of GRP78 enhances adenosine-induced apoptosis in a caspase-4-dependent manner in HepG2 cells (Figures 3 and 4). Taken together, these findings first indicate that GRP78 may play a decisive role on cell survival in adenosine-induced apoptosis and knockdown GRP78 sensitizes adenosine cytotoxicity in HepG2 cells.

Our data also imply that ER stress-induced apoptosis occurs in part via activation of caspase-4. Because caspase-4 knockdown cannot completely block cell apoptosis and prevent the loss of ΔΨm, the existence of parallel casapse-4-independent apoptotic pathways is suggested. Adenosine causes cytosolic Ca^2+^ overload, ΔΨm loss and caspase-3 activation. Knockdown of GRP78 further aggravates the loss of ΔΨm and adenosine-induced apoptosis, indicating that other apoptotic pathways such as the mitochondrion pathway, may participate in the process [37] and that GRP78 is involved in the regulation of ΔΨm [30,31]; other possibilities include autophagy [38] and other miRNAs, such as miR-30d, miR-181a and miR-199a-5p [39].

## 3. Materials and Methods

### 3.1. Reagents and Antibodies

Fetal bovine serum (FBS), Dulbecco’s modified Eagle’s medium (DMEM), penicillin, streptomycin, Lipofectamine 2000, adenovirus, plasmid purification kits, Gateway™ Adenovirus Cloning Kit and trypsin-EDTA were obtained from Invitrogen (Shanghai, China). RIPA buffer and protease inhibitor cocktail were purchased from Sigma-Aldrich (St. Louis, MO, USA). The real-time PCR kit was obtained from Tiangen Biotech Inc. (Beijing, China), CCK-8 Kit purchased from Beyotime Biotech Inc. (Haimen, Jiangsu, China), and DAPI and the DeadEndTM Colorimetric TUNEL System was purchased from Promega Corporation (Madison, WI, USA). An enhanced chemiluminescence detection reagent kit (ECL kit) was purchased from Thermo Scientific (Waltham, MA, USA). Primary antibodies against β-actin, Bax, Bad, m-calpain, caspase-4, cleaved and pro-caspase-3, and CHOP were obtained from Santa Cruz Biotechnology (Santa Cruz, CA, USA). Avidin-biotin-horseradish peroxidase (ABC) complex, bovine serum albumin (BSA), horseradish-peroxidase (HRP)-conjugated goat anti-rabbit and anti-mouse secondary antibody were obtained from Shanghai Sangon (Shanghai, China). For experiments, 10 mmol/L adenosine was dissolved in DMEM; 2% BSA, 4% paraformaldehyde and 0.2% triton X-100 were dissolved in phosphate-buffered saline (PBS).

### 3.2. Cell Culture and Experimental Groups

HepG2 and 293A cells, obtained from the American Type Culture Collection, were cultured in DMEM supplemented with 10% (*v*/*v*) fetal bovine serum, penicillin (final concentration, 100 U/mL), and streptomycin (final concentration, 0.1 mg/mL), under a humidified atmosphere of 5% CO_2_ and 95% air at 37 °C. The culture medium was changed every other day. Cells were passaged at 70%–80% confluence. For the TUNEL assay and DAPI staining, cells were cultured on cover slips in DMEM for 24 h before adenosine treatment.

For RNAi experiments, HepG2 cells were transfected with adenovirus encoding a siRNA targeted against GRP78 (Ad-shGRP78) or an empty adenovirus vector only expressing GFP (Ad-GFP). After a change of fresh medium 24 h later, the transfected cells were incubated with or without 4.0 mmol/L adenosine in complete medium for a further 24 h, then the cells were collected. For the negative control, cells were transfected with empty vector. The positive control was cells transfected with empty vector and treated with adenosine (Ado).

### 3.3. Construction of the Recombinant Adenovirus Vector and Adenovirus Transfection

The pcDNA6.2-GW/EmGFP-GRP78-siR plasmid and pcDNA6.2-GW/EmGFP-Caspase-4-siR plasmid vectors were constructed as previously described [40]. Briefly, for pcDNA6.2-GW/EmGFP-GRP78- siR, four siRNA candidate sequences targeting GRP78 (GRP78-1 to GRP78-4) were cloned into pcDNA6.2-GW/emerald green fluorescent protein (GFP) vector. A GFP-expressing empty vector served as a control. The sequences that had the best interference efficiency in 293A cells (GRP78-1), “Forward: 5′-TGCTGACCAGTTGCTGAATCTTTGGAGTTTTGGCCACTGACTGACTCCAAAGA CAGCAACTGGT-3′; Reverse: 5′-CCTGACCAGTTGCTGTCTTTGGAGTCAGTCAGTGGCCAA AACTCCAAAGATTCAGCAACTGGTC-3′” were chosen and co-transfected with GRP78 vector for real-time PCR and western blot assays. An LR recombination reaction was performed between the cloned plasmid and pAd/CMV/V5-DEST™ destination vector by using LR Clonase™ enzyme mix, and the reaction mixture was transformed into competent DH5α bacteria, and the true expression clones were selected as spectinomycin-resistant. The success of shGRP78 or GFP insertion into adenoviral plasmid was confirmed by DNA sequencing. Adenoviral vectors carrying either shGRP78 (Ad-shGRP78) or GFP (Ad-GFP) were then linearized by *Pac*I digestion and used to transfect 293A cell using Lipofecfamine 2000 reagent. Transfected cells were selected as blasticidin-resistant and the transfection efficiency was monitored by fluoresence microscopy. The viral titer was determined in a 96-well plate according to the manufacturer’s instructions. A multiplicity of infection (MOI) of 1 was defined as the amount of adenoviral vector necessary to positively transfect 100% of cells after 48 h as determined by GFP fluorescence.

For the pcDNA6.2-GW/EmGFP-Caspase-4-siR plasmid vector, the effective oligo sequences were “Forward: 5′-TGCTGTCATCATGCACAGTTCCGCAGGTTTTGGCCACTGACTGACCTGCGGAA GTGCATGATGA-3′, Reverse 5′-CCTGTCATCATGCACTTCCGCAGGTCAGTCAGTGGCCAA AACCTGCGGAACTGTGCATGATGAC-3′”. For transfection, 1 × 10^5^ cells were plated in six-well plates and transfected with the p-shCasp-4 plasmid vectors according to the manufacturer’s instructions.

### 3.4. Determination of Cell Viability by CCK-8 Assay

HepG2 cell viability was evaluated using the Cell Counting Kit-8 (CCK-8). Cells were inoculated into 96-well plates at 5 × 10^5^ cells per well and grown for 24 h. HepG2 cells were transfected with adenovirus and divided into different groups: Ad-GFP group (control) and Ad-shGRP78 groups (smallRNA interference, adenovirus titer at multiplicities of infection (MOIs) of 20, 50, 100 and 200). After a change of fresh medium 24 h later, cells were treated with or without 4.0 mmol/L adenosine for another 24 h. 10 μL CCK-8 (5.0 g/L) per well was added into the medium and cells were incubated at 37 °C for another 4 h in a 5% CO_2_ incubator. The absorbance (*A*) of each well was measured with a microtiter plate reader (KHB Labsystems Wellscan K3, Finland) at a wavelength of 450 nm. Survival rate (%) = (*A*_sample_ − *A*_untreated_)/(*A*_control_ − *A*_untreated_). The experiment was repeated three separate times.

### 3.5. Cell Cycle Phase Determination by Flow Cytometry

HepG2 cells were inoculated into 6-well plates at 5 × 10^5^ cells per well, cultured overnight and transfected with Ad-GFP or Ad-shGRP78 for 24 h, then treated with or without adenosine for another 24 h. Cells were washed with 5 mL PBS (pH 7.2), and fixed in 70% ethanol at 4 °C overnight. Cells were washed twice with PBS to remove the ethanol and passed through a 0.44-mm filter to remove aggregates. The cells were incubated in PBS containing RNase A (2.0 μg/mL) for 1 h at 37 °C, followed by staining with PI (5.0 μg/mL) for 20 min while protected from light on ice. Cells were collected on a nylon mesh filter (pore size, 40 μm) and cell cycle phases including the sub-G1 phase (apoptotic cells) were assayed by a flow cytometer (FACSCalibur, Becton-Dickinson, San Jose, CA, USA) at an excitation of 488 nm and an emission of 585 nm, and analyzed using ModFit software (Verity Software Inc., Topsham, ME, USA). The experiment was repeated three separate times.

### 3.6. Identification of Cell Apoptosis by DAPI Staining and TUNEL Assay

To observe cells undergoing apoptosis, DAPI staining was performed. Cells were fixed in 4% paraformaldehyde for 30 min at room temperature and then permeabilized with 0.2% Triton X-100 in PBS for 5 min at room temperature. DAPI (500 ng/mL) staining was performed at room temperature for 10 min. The morphology of the nuclei was viewed and captured with a fluorescence microscope (Olympus BX51, Tokyo, Japan).

To obtain further evidence for apoptosis, DNA fragmentation was also determined using a TdT-mediated dUTP-FITC nick-end labeling (TUNEL) assay as described previously [7,9]. Cells were fixed by immersing slides in freshly prepared 4% methanol-free formaldehyde solution in PBS for 20 min at room temperature and permeabilized with 0.2% Triton X-100 for 5 min. Cells were labeled with fluorescein TUNEL reagent mixture for 60 min at 37 °C according to the manufacturer’s instructions. After immersing the slides in 2 × SSC for 15 min at room temperature, endogenous peroxidases were blocked. Slides were then incubated with horseradish-peroxidase-labeled streptavidin (HRP) solution for 30 min. Finally, slides were incubated with diaminobenzidine (DAB) components for 10 min and examined under a light microscope. TUNEL-positive (brown staining) cells were defined as apoptotic cells.

### 3.7. Measurement of Intracellular Ca^2+^ Concentration

The levels of Ca^2+^ concentration [Ca^2+^]i were monitored using Fluo-3/AM fluorescence assay by laser scanning confocal microscopy. Briefly, Fluo-3/AM was initially dissolved in DMSO and stored at −20 °C. Cells were incubated with 20 μmol/L Fluo-3/AM at 37 °C for 1 h. After incubation, cells were washed with normal Tyrode’s solution to remove the extracellular Fluo-3/AM, then cells were excited at 488 nm through an eclipse microscope (Nikon E-600, Tokyo, Japan) built in a laser confocal scanning system (ACAS Ultima 312, Meridian Instruments, Freeland, WA, USA). Emitted fluorescence intensity was recorded at 530 nm and 10–40 nm pinhole. The changes in [Ca^2+^]i are presented as background-subtracted normalized fluorescence (Δ*F*/*F*_0_) where *F* is the fluorescence intensity, *F*_0_ is the resting fluorescence recorded under steady-state conditions at the beginning of an experiment, and Δ*F* = *F* − *F*_0_. The fluorescence intensity of the pixel was collected and managed with the software of the instrument.

### 3.8. Detection of Mitochondrial Membrane Potential (ΔΨm) by Flow Cytometric Analysis

Rhodamine 123 (Rh123) is a fluorescent cationic dye that binds to the polarized mitochondrial membrane and accumulates as aggregates in the mitochondria of normal cells. Rh123 was prepared in ethanol as a 5 mg/mL stock solution. At the end of the reaction time, the cells were harvested and washed twice with ice-cold PBS by centrifugation at 1000 rpm for 10 min, then 0.5 mL PBS containing 10 μg/mL Rh123 was added to the cells. The tubes were vortexed gently and incubated at 37 °C in the dark for 30 min. The Rh123 staining intensity was captured using a fluorescence microscope. Intensity of Rh123 is directly related to mitochondrial membrane potential (ΔΨm). The changes in mitochondrial membrane potential were monitored by a flow cytometer (FACSCalibur, Becton-Dickinson, Franklin Lakes, NJ, USA) at an excitation of 488 nm and an emission of 585 nm.

### 3.9. RNA Preparation and Real-Time PCR Assay

HepG2 total RNA was isolated from cultured cells using the Trizol reagent (Invitrogen, Carlsbad, CA, USA), according to the manufacturer’s instructions. For reverse transcriptase analysis, 1 μg of total RNA was reversely transcribed using a Revert Aid First Strand cDNA Synthesis Kit (Fermentas, Shenzhen, China). Real-time PCR amplification with one microliter of the reverse transcriptase reaction mixture was performed with SYBR Green Real-time PCR MasterMix-Plus- (Toyobo, Japan). The initial denaturation step was 95 °C for 1 min followed by 95 °C for 15 s, 58 °C for 15 s, and 72 °C for 45 s for a total 40 cycles of amplification. Primer sequences were designed using Primer Premier 5 software (Premier Inc., Toronto, ON, Canada) and the conditions for PCR amplification were adjusted for the targeted genes (Table 1). 18s rRNA was used as an internal control. After PCR cycling, 2 μL of PCR products were subjected to agarose gel electrophoresis and quantified by capturing the ethidium bromide absorbance with a fluorescence microscope (Olympus BX51, Tokyo, Japan). Primer Premier 5 software was used to analyze the data and calculate *C*t (threshold cycle) values. The targeted genes and 18s rRNA transcript levels were estimated using the formula 2^−ΔΔ^*^C^*^t^ where ΔΔ*C*t represents the difference in *C*t values between target and housekeeping assays. To confirm the specificity of amplification, melting curve analysis was carried out after the final cycle of amplification. All samples were performed in triplicate, and the experiment was repeated three separate times.

### 3.10. Protein Extraction and Western Blot Analysis

HepG2 cells were washed with ice-cold PBS, harvested and lysed in RIPA buffer (10 mM Tris-HCl pH 8.0, 1% sodium deoxycholate, 0.1% SDS, 1% Triton X-100, 140 mmol/L NaCl, 0.5% NP-40 and 1 mmol/L PMSF). Cell lysates were centrifuged at 12,000 rpm at 4 °C for 10 min. The supernatant was boiled for 5 min and subjected to 12% sodium dodecylsulfate polyacrylamide gel electrophoresis (SDS-PAGE) and transferred to a nitrocellulose membrane. The membranes were blocked with 5% fat-free milk for 1 h at room temperature, followed by incubation with primary antibodies against β-actin (1:800), m-calpain (1:400), Bax (1:400), Bak (1:400), caspase-4 (1:400), total caspase-3 (1:400), cleaved caspase-3 (1:400) and CHOP (1:400) at 4 °C overnight. The next day, the membranes were incubated with biotin-conjugated secondary antibody for 1 h at room temperature, followed by incubation with an avidin-biotin-horseradish peroxidase (HRP) complex at room temperature for 30 min. Bands were visualized by an ECL detection system (Thermo Scientific, Waltham, MA, USA). Protein expression was analyzed by the Quantity One software (Bio-Rad, Hercules, CA, USA) and normalized to that of β-actin.

### 3.11. Statistical Analysis

Data are expressed as the mean ± SD of at least three independent experiments. Results were analyzed with the unpaired Student’s *t*-test or one way ANOVA for different groups in SPSS 17.0 (SPSS Inc, Chicago, IL, USA). A value of *p* < 0.05 was considered as statistically significant, and *p* < 0.01 was considered as remarkable statistical significance.

## 4. Conclusions

In the current study, our results demonstrate that knockdown GRP78 enhances HepG2 cell sensitivity to adenosine by causing G0/G1 arrest and stimulating Bax and Bak protein levels, cytosolic Ca^2+^ overload and loss of ΔΨm. Activated CHOP and caspase-4 signal pathways further cleave procaspase-3 into active caspase-3 and induce apoptosis. GRP78 may play a decisive role on the survival of HepG2 cells in adenosine-induced ER stress.

## Figures and Tables

**Table 1.** Sequences of primers used for real-time PCR.

Primer name	Primer sequence
GRP78- sense	5′-CCTGGTACTGCTTGATGTAT-3′
GRP78-antisense	5′-TTCTGCTGTATCCTCTTCAC-3′
CHOP-sense	5′-CTTCATACATCACCACACCT-3′
CHOP-antisense	5′-GTAGTCAGTAGCCACTTCTG-3′
caspase-3-sense	5′-CTATTGTGAGGCGGTTGT-3′
caspase-3-antisense	5′-TCCAGAGTCCATTGATTCG-3′
caspase-4-sense	5′-TGAACTGGAAGGAAGAGGAA-3′
caspase-4-antisense	5′-GCGGTTGTTGAATATCTGGA-3′
18s rRNA-sense	5′-TGGTGGAGCGATTTGTCTG-3′
18s rRNA-antisense	5′-AATGGGGTTCAACGGGTTAC-3′

## Abbreviations

GFP: green fluorescent protein
MOI: multiplicity of infection
Ad-GFP: an only expressing GFP empty adenovirus vector
Ad-shGRP78: recombinant adenovirus carrying short hairpin RNA targeted against GRP78
p-GFP: a plasmid vector carrying GFP
p-shCasp-4: a plasmid vector carrying short hairpin RNA targeted against caspase-4

## References

[1] Cao H., Phan H., Yang L.X. (2012). Improved chemotherapy for hepatocellular carcinoma. Anticancer Res.

[2] Bruix J., Sherman M. (2011). Management of hepatocellular carcinoma: An update. Hepatology.

[3] Lee A.S. (2007). GRP78 induction in cancer: Therapeutic and prognostic implications. Cancer Res.

[4] Gorman A.M., Healy S.J., Jager R., Samali A. (2012). Stress management at the ER: Regulators of ER stress-induced apoptosis. Pharmacol. Ther.

[5] Saito M., Yaguchi T., Yasuda Y., Nakano T., Nishizaki T. (2010). Adenosine suppresses CW2 human colonic cancer growth by inducing apoptosis via A(1) adenosine receptors. Cancer Lett.

[6] Wu L.F., Li G.P., Feng J.L., Pu Z.J. (2006). Molecular mechanisms of adenosine-induced apoptosis in human HepG2 cells. Acta Pharmacol. Sin.

[7] Wu L.F., Wei B.L., Guo Y.T., Ye Y.Q., Li G.P., Pu Z.J., Feng J.L. (2012). Apoptosis induced by adenosine involves endoplasmic reticulum stress in EC109 cells. Int. J. Mol. Med.

[8] Shirali S., Aghaei M., Shabani M., Fathi M., Sohrabi M., Moeinifard M. (2013). Adenosine induces cell cycle arrest and apoptosis via cyclinD1/Cdk4 and Bcl-2/Bax pathways in human ovarian cancer cell line OVCAR-3. Tumor Biol.

[9] Wu L.F., Ye Y.Q., Huang G.Y., Li H.B., Li G.P., Pu Z.J., Wei B.L., Feng J.L. (2011). Involvement of endoplasmic reticulum stress in adenosine-induced human hepatoma HepG2 cell apoptosis. Oncol. Rep.

[10] Li J., Lee A.S. (2006). Stress induction of GRP78/BiP and its role in cancer. Curr. Mol. Med.

[11] Hetz C. (2012). The unfolded protein response: Controlling cell fate decisions under ER stress and beyond. Nat. Rev. Mol. Cell Biol.

[12] Woehlbier U., Hetz C. (2011). Modulating stress responses by the UPRosome: A matter of life and death. Trends Biochem. Sci.

[13] Wu J., Kaufman R.J. (2006). From acute ER stress to physiological roles of the Unfolded Protein Response. Cell Death Differ.

[14] Roller C., Maddalo D. (2013). The molecular chaperone GRP78/BiP in the development of chemoresistance: Mechanism and possible treatment. Front. Pharmacol.

[15] Hitomi J., Katayama T., Eguchi Y., Kudo T., Taniguchi M., Koyama Y., Manabe T., Yamagishi S., Bando Y., Imaizumi K. (2004). Involvement of caspase-4 in endoplasmic reticulum stress-induced apoptosis and Abeta-induced cell death. J. Cell Biol.

[16] Szegezdi E., Logue S.E., Gorman A.M., Samali A. (2006). Mediators of endoplasmic reticulum stress-induced apoptosis. EMBO Rep.

[17] Pyrko P., Schonthal A.H., Hofman F.M., Chen T.C., Lee A.S. (2007). The unfolded protein response regulator GRP78/BiP as a novel target for increasing chemosensitivity in malignant gliomas. Cancer Res.

[18] Uckun F.M., Qazi S., Ozer Z., Garner A.L., Pitt J., Ma H., Janda K.D. (2011). Inducing apoptosis in chemotherapy-resistant B-lineage acute lymphoblastic leukaemia cells by targeting HSPA5, a master regulator of the anti-apoptotic unfolded protein response signalling network. Br. J. Haematol.

[19] Scriven P., Coulson S., Haines R., Balasubramanian S., Cross S., Wyld L. (2009). Activation and clinical significance of the unfolded protein response in breast cancer. Br. J. Cancer.

[20] Pootrakul L., Datar R.H., Shi S.R., Cai J., Hawes D., Groshen S.G., Lee A.S., Cote R.J. (2006). Expression of stress response protein Grp78 is associated with the development of castration-resistant prostate cancer. Clin. Cancer Res.

[21] Dong D., Stapleton C., Luo B., Xiong S., Ye W., Zhang Y., Jhaveri N., Zhu G., Ye R., Liu Z. (2011). A critical role for GRP78/BiP in the tumor microenvironment for neovascularization during tumor growth and metastasis. Cancer Res.

[22] Roue G., Perez-Galan P., Mozos A., Lopez-Guerra M., Xargay-Torrent S., Rosich L., Saborit-Villarroya I., Normant E., Campo E., Colomer D. (2011). The Hsp90 inhibitor IPI-504 overcomes bortezomib resistance in mantle cell lymphoma *in vitro* and *in vivo* by down-regulation of the prosurvival ER chaperone BiP/Grp78. Blood.

[23] Krol J., Loedige I., Filipowicz W. (2010). The widespread regulation of microRNA biogenesis, function and decay. Nat. Rev. Genet.

[24] Virrey J.J., Dong D., Stiles C., Patterson J.B., Pen L., Ni M., Schonthal A.H., Chen T.C., Hofman F.M., Lee A.S. (2008). Stress chaperone GRP78/BiP confers chemoresistance to tumor-associated endothelial cells. Mol. Cancer Res.

[25] Yung H.W., Charnock-Jones D.S., Burton G.J. (2011). Regulation of AKT phosphorylation at Ser473 and Thr308 by endoplasmic reticulum stress modulates substrate specificity in a severity dependent manner. PLoS One.

[26] Wu L.F., Li G.P., Su J.D., Pu Z.J., Feng J.L., Ye Y.Q., Wei B.L. (2010). Involvement of NF-κB activation in the apoptosis induced by extracellular adenosine in human hepatocellular carcinoma HepG2 cells. Biochem. Cell Biol.

[27] Wang N.S., Unkila M.T., Reineks E.Z., Distelhorst C.W. (2001). Transient expression of wild-type or mitochondrially targeted Bcl-2 induces apoptosis, whereas transient expression of endoplasmic reticulum-targeted Bcl-2 is protective against Bax-induced cell death. J. Biol. Chem.

[28] Foyouzi-Youssefi R., Arnaudeau S., Borner C., Kelley W.L., Tschopp J., Lew D.P., Demaurex N., Krause K.H. (2000). Bcl-2 decreases the free Ca2+ concentration within the endoplasmic reticulum. Proc. Natl. Acad. Sci. USA.

[29] Scorrano L., Oakes S.A., Opferman J.T., Cheng E.H., Sorcinelli M.D., Pozzan T., Korsmeyer S.J. (2003). BAX and BAK regulation of endoplasmic reticulum Ca2+: A control point for apoptosis. Science.

[30] He H., Lam M., McCormick T.S., Distelhorst C.W. (1997). Maintenance of calcium homeostasis in the endoplasmic reticulum by Bcl-2. J. Cell Biol.

[31] Pizzo P., Pozzan T. (2007). Mitochondria-endoplasmic reticulum choreography: Structure and signaling dynamics. Trends Cell Biol.

[32] Bromati C.R., Lellis-Santos C., Yamanaka T.S., Nogueira T.C., Leonelli M., Caperuto L.C., Gorjao R., Leite A.R., Anhe G.F., Bordin S. (2011). UPR induces transient burst of apoptosis in islets of early lactating rats through reduced AKT phosphorylation via ATF4/CHOP stimulation of TRB3 expression. Am. J. Physiol. Regul. Integr. Comp. Physiol.

[33] Brunelle J.K., Letai A. (2009). Control of mitochondrial apoptosis by the Bcl-2 family. J. Cell Sci.

[34] Hetz C., Bernasconi P., Fisher J., Lee A.H., Bassik M.C., Antonsson B., Brandt G.S., Iwakoshi N.N., Schinzel A., Glimcher L.H. (2006). Proapoptotic BAX and BAK modulate the unfolded protein response by a direct interaction with IRE1alpha. Science.

[35] Morishima N., Nakanishi K., Takenouchi H., Shibata T., Yasuhiko Y. (2002). An endoplasmic reticulum stress-specific caspase cascade in apoptosis. Cytochrome c-independent activation of caspase-9 by caspase-12. J. Biol. Chem.

[36] Lakshmanan U., Porter A.G. (2007). Caspase-4 interacts with TNF receptor-associated factor 6 and mediates lipopolysaccharide-induced NF-kappaB-dependent production of IL-8 and CC chemokine ligand 4 (macrophage-inflammatory protein-1). J. Immunol.

[37] Tamura K., Kanno T., Fujita Y., Gotoh A., Nakano T., Nishizaki T. (2012). A(2a) adenosine receptor mediates HepG2 cell apoptosis by downregulating Bcl-X(L) expression and upregulating Bid expression. J. Cell. Biochem.

[38] Cook K.L., Shajahan A.N., Wärri A., Jin L., Hilakivi-Clarke L.A., Clarke R. (2012). Glucose-regulated protein 78 controls cross-talk between apoptosis and autophagy to determine antiestrogen responsiveness. Cancer Res.

[39] Su S.F., Chang Y.W., Andreu-Vieyra C., Fang J.Y., Yang Z., Han B., Lee A.S., Liang G. (2013). miR-30d, miR-181a and miR-199a-5p cooperatively suppress the endoplasmic reticulum chaperone and signaling regulator GRP78 in cancer. Oncogene.

[40] Huang G., Zeng Y., Liang P., Zhou C., Zhao S., Huang X., Wu L., He X. (2012). Indoleamine 2,3-Dioxygenase (IDO) Downregulates the Cell Surface Expression of the CD4 Molecule. Int. J. Mol. Sci.

